# CNpare: matching DNA copy number profiles

**DOI:** 10.1093/bioinformatics/btac371

**Published:** 2022-05-31

**Authors:** Blas Chaves-Urbano, Barbara Hernando, Maria J Garcia, Geoff Macintyre

**Affiliations:** Computational Oncology Group, Spanish National Cancer Research Centre (CNIO), 28029 Madrid, Spain; Computational Oncology Group, Spanish National Cancer Research Centre (CNIO), 28029 Madrid, Spain; Computational Oncology Group, Spanish National Cancer Research Centre (CNIO), 28029 Madrid, Spain; Computational Oncology Group, Spanish National Cancer Research Centre (CNIO), 28029 Madrid, Spain

## Abstract

**Summary:**

Selecting the optimal cancer cell line for an experiment can be challenging given the diversity of lines available. Here, we present CNpare, which identifies similar cell line models based on genome-wide DNA copy number.

**Availability and implementation:**

CNpare is available as an R package at https://github.com/macintyrelab/CNpare. All analysis performed in the manuscript can be reproduced via the code found at https://github.com/macintyrelab/CNpare_analyses.

**Supplementary information:**

Supplementary data are available at *Bioinformatics* online.

## 1 Introduction

**Fig. 1.**
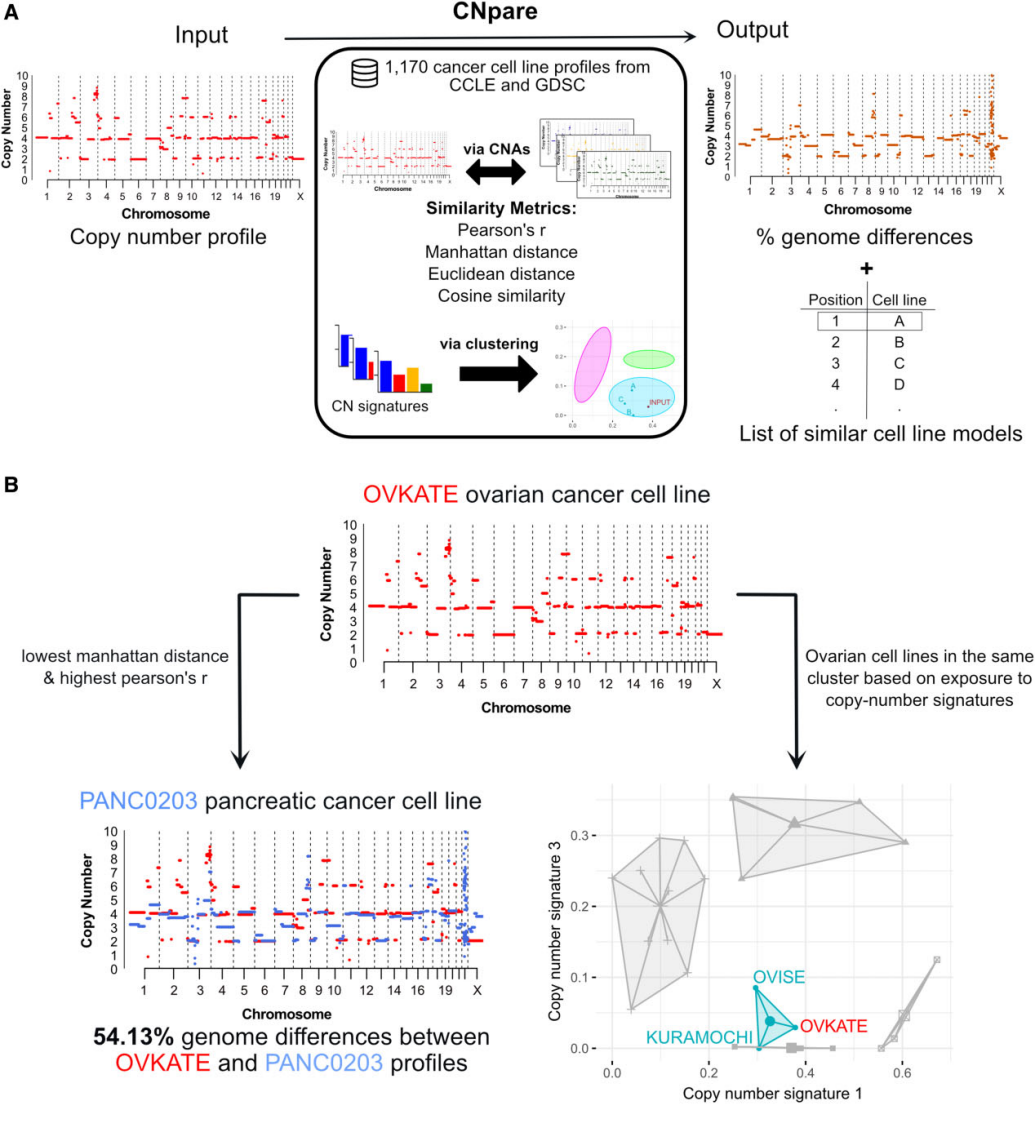
Overview of CNpare and an example of a cell line match. (**A**) Schematic providing a high-level overview of CNpare’s workflow and computation. The user inputs an absolute copy number profile (left) and CNpare compares this to a precomputed database of cell line copy number from the CCLE and GDSC projects, using a series of different comparison metrics (center). Output is in the form of a list of cell lines ranked based on the strength of match to the input profile. Included in the output is a graphical representation of differences between the genomes and an estimate on the percentage genome difference. (**B**) Example of a CNpare workflow using OVKATE cell line as the input cell line. The top plot shows the copy number profile of the OVKATE ovarian cancer cell line used as input to CNpare. On the left side, the copy number profile of the top hit found using Pearson’s *r* and Manhattan distance is displayed in blue, along with the OVKATE line in red. Underneath the percentage genome difference between the profiles is listed. On the right-hand side, the results of matching the OVKATE cell line based on copy number signatures are displayed, showing the results of clustering all ovarian cancer cell lines based on seven copy number signatures (Macintyre *et al.*, 2018). For visualization purposes, the two signatures with the highest variation across cluster means are shown. Each large dot represents the cluster centroid and each small dot represents a cell line. The cluster containing the OVKATE cell line is indicated (A color version of this figure appears in the online version of this article.)

Immortalized cancer cell lines are an integral part of cancer research and the development of new therapies (Drost and Clevers, 2018; Sharma *et al.*, 2010). Cell lines are often selected based on their tissue of origin. However, new approaches are available that facilitate appropriate cell line selection based on molecular similarities such as gene expression, DNA methylation and genomics (Ben-David *et al.*, 2018; Dancik *et al.*, 2011; Domcke *et al.*, 2013; Mohammad *et al.*, 2019; Najgebauer *et al.*, 2020; Peng *et al.*, 2021; Salvadores *et al.*, 2020; Sinha *et al.*, 2015; Vincent *et al.*, 2015; Warren *et al.*, 2021; Yu *et al.*, 2019; Zhao *et al.*, 2017). A subset of these approaches perform DNA copy number-based comparison at different resolutions including gene-level copy number, chromosome arm copy number, ploidy or genome doubling status (Ben-David *et al.*, 2018; Domcke *et al.*, 2013; Najgebauer *et al.*, 2020; Zhao *et al.*, 2017). However, a specific tool for computing similarity based on genome-wide copy number is lacking. Here, we present CNpare, which identifies similar cell line models based on genome-wide DNA copy number. CNpare compares copy number profiles using four different similarity metrics, quantifies the extent of genome differences between pairs and facilitates comparison based on copy number signatures (Macintyre *et al.*, 2018) (Fig. 1A).

## 2 Materials and methods

CNpare is designed to compare and contrast genome-wide cancer cell line copy number profiles. The user inputs one or more genome-wide absolute copy number profiles in the form of a segment table (chromosome, start, end, copy number) and these are compared to a precomputed database of cancer cell line profiles. This includes profiles of 1170 human cancer cell lines from the Cancer Cell Line Encyclopaedia (CCLE) (Ghandi *et al.*, 2019) project and the Genomics of Drug Sensitivity in cancer (GDSC) project (Yang *et al.*, 2013). Copy number profiles from these cancer cell lines were generated using ASCAT (Van Loo *et al.*, 2010).

To facilitate comparison between two profiles, *segment tables* are converted to *bin tables* where the copy number is reported for evenly sized bins across the genome (default 500 kb). To adjust for noise at copy number boundaries, a window-based smoothing procedure is used to align boundaries across samples (see Supplementary Material).

Tools that compare genome-wide copy number between tumors from the same patient (Schwarz *et al.*, 2014) or cells within a tumor (Salehi *et al.*, 2021), rely on evolutionary models. However, tumors from different patients do not have a shared evolutionary relationship, therefore more traditional similarity metrics can be used. Thus, similarity between the bin-level copy number of two profiles is computed using different metrics: Pearson correlation, Manhattan distance, Euclidean distance and Cosine similarity. Depending on the metric used, emphasis can be placed on different properties of the copy number profile (summarized in Supplementary Table S1). CNpare also computes the percentage genome difference between profiles and provides a visual representation of the differences.

In addition to direct comparison of copy number profiles, CNpare can also be used to perform comparison using copy number signatures (Macintyre *et al.*, 2018). Copy number signatures provide a readout of the different types of chromosomal instability (CIN) that generated the copy number profile. Therefore, this type of comparison allows cell lines to be compared based on the mutational processes present in the sample, rather than the output of these processes. This comparison is also extended to clusters of cell lines with similar patterns of genomic alterations determined via k-means clustering and visualized using the two signatures with the highest variability across clusters.

## 3 Results

We assessed the performance of CNpare in a controlled setting (matching profiles from separate cultures of the same cell lines), and in real-world scenarios (finding the nearest match for cell lines and assessing suitability).

### 3.1 Assessing performance using matched cell line cultures

We used separate cultures of the same cell lines profiled as part of the CCLE and GDSC projects (304 pairs) and observed the ability of CNpare to identify, for each GDSC line, the correctly matched line in the CCLE database (Supplementary Fig. S1). In 100% of cases, the top hit for each GDSC line was the matched line in the CCLE database. Eighty-seven percent (265 cells) were matched correctly across all similarity metrics, with the remaining 13% being matched correctly only by Pearson correlation and Cosine similarity (not Manhattan and Euclidean distance). For these unmatched cases, the ploidy estimated by ASCAT differed between the CCLE and GDSC cultures, causing comparison based on Manhattan and Euclidean distances to return the wrong cell line. As Pearson correlation and Cosine similarity are normalized measures, they successfully identified the correct profile independent of difference in ploidy status between the cultures. This difference in metric performance demonstrates the choice of metric can determine whether the comparison is ploidy aware or agnostic. As such, we developed a guide on which metrics to use for different circumstances (Supplementary Table S1). Similar performance was observed across different bin sizes (Supplementary Table S2), even after introducing noise in up to 50% of the copy number profile (Supplementary Methods and Supplementary Fig. S2).

To compare against alternative approaches, we computed gene-based copy number, chromosome arm-based copy number, ploidy and gene expression for all cell lines in the database. Using these data to compare the cell line culture pairs, we found that gene-based copy number matched 63% of the cell lines correctly, arm-based copy number 91%, ploidy 43% and gene expression 38% (Supplementary Data S1). This suggests that high-resolution DNA copy number-based matching provides a robust method of identifying similar cell line models.

### 3.2 Assessing performance using real-world scenarios

For each cell line in the CCLE database, we sought its nearest match using CNpare and observed whether this match was significant (the observed correlation measure was greater than expected by chance) or non-significant (a random match). Using Pearson’s *r*, 93.9% (567/604) of the matched cell lines were significant. Using Manhattan’s distance 41.6% (251/604) were significant. This suggests that a good, ploidy agnostic match can nearly always be found using CNpare; however, the current database only supports good, ploidy aware matches ∼40% of the time.

We also explored a specific match scenario using the high-grade serous ovarian cancer cell line OVKATE. First, we directly compared the OVKATE copy number profile with copy number profiles in the database. The cell line which had the most similar profile was PANC0203 (Pearson’s *r*= 0.42, p-value = 0.02, with 54.13% genome difference, Fig. 1B). Despite this line being derived from a pancreatic adenocarcinoma, it showed highly correlated gene expression (Pearson’s *r* = 0.43, p-value = 0.02: Supplementary Fig. S3A) suggesting a robust match. Second, we sought a tissue matched line by comparing copy number signatures. We performed clustering of the OVKATE line with all ovarian cancer cell lines based on copy number signatures using cosine similarity, which allowed us to identify the ovarian cancer cell lines with similar mutational mechanisms regardless of their specific copy number changes. OVKATE clustered with two other ovarian cell lines OVISE and KURAMOCHI (Fig. 1B and Supplementary Fig. S4A and B). These lines both showed correlated gene expression (Pearson‘s *r* = 0.38, p-value = 0.03; and Pearson‘s *r*= 0.48, p-value = 0.01; Supplementary Fig. S3B and C) suggesting a relevant match in terms of CIN patterns and resulting gene expression changes.

## 4 Discussion

Here, we present CNpare, a cell line copy number profile comparison tool for the purpose of selecting optimal cell line models. CNpare is the first stand-alone tool to facilitate comparison of cell line models based on high-resolution, genome-wide copy number. This complements existing approaches based on low-resolution copy number, gene expression and methylation (Ben-David *et al.*, 2018; Dancik *et al.*, 2011; Domcke *et al.*, 2013; Mohammad *et al.*, 2019; Najgebauer *et al.*, 2020; Peng *et al.*, 2021; Salvadores *et al.*, 2020; Vincent *et al.*, 2015; Warren *et al.*, 2021; Yu *et al.*, 2019; Zhao *et al.*, 2017). In addition, CNpare offers the option of comparing copy number profiles from a more functional point of view by using copy number signatures.

CNpare can also be applied to other settings including: quality control—ensuring the sequenced copy number profile of a cell line matches the reference profile; assessing differences between cell line cultures—by estimating the percentage genome difference; and finding the best cell line model for a tumor profile—based on copy number profiles or copy number signatures.

Despite showing excellent performance during benchmarking, this tool has two key limitations: (i) resolution—comparison is made at 500 kb resolution (default bin size). While it is possible to increase the resolution up to 30 kb, resolution beyond this is limited as the SNP6 technology underpinning the database does not facilitate higher resolution; (ii) total absolute copy number as input. Total absolute copy number is currently required input and performance is dependent on the accuracy of the method used to compute it. We recommend using ASCAT (Van Loo *et al.*, 2010) as it matches the method used across the database.

Despite these limitations (which may be resolved over time as additional data becomes available), our results demonstrate that CNpare can be used to select appropriately matched cancer cell line models, providing a valuable tool for improving cancer research in the context of studying CIN.

## Supplementary Material

btac371_Supplementary_Data
