# Supplementary material for: CNpare: matching DNA copy number profiles
Source: Bioinformatics. 2022 May 31;38(14):3638–41. doi: 10.1093/bioinformatics/btac371 (PMC9272807; doi:10.1093/bioinformatics/btac371)
Supplement: btac371_Supplementary_Data [file btac371_supplementary_data.zip › btac371-suppl_data/Supplementary Material.pdf]

SUPPLEMENTARY MATERIAL

Table of contents

Supplementary figures and tables **2**
- Supplementary Figure 1 2
- Supplementary Figure 2 3
- Supplementary Figure 3 4
- Supplementary Figure 4 4
- Supplementary Table 1 5
- Supplementary Table 2 5

Supplementary methods **5**
- Data acquisition and curation 5
- Data preprocessing 6
- Comparison of copy number profiles 6
- Calculating percentage genome difference 6
- Robustness analysis 7
  - Assessing performance across different bin sizes 7
  - Assessing performance with noise via simulations 7
- Comparing performance to other approaches 7
  - Gene-level copy number 7
  - Chromosome arm copy number 8
  - Ploidy status 8
  - Gene-expression profiles 8
- Real world performance assessment 8
  - Next best matches for each cell line in database 8
  - Testing suitability of OVKATE cell line matches 8
- Software versions 8

References **10**

Supplementary figures and tables

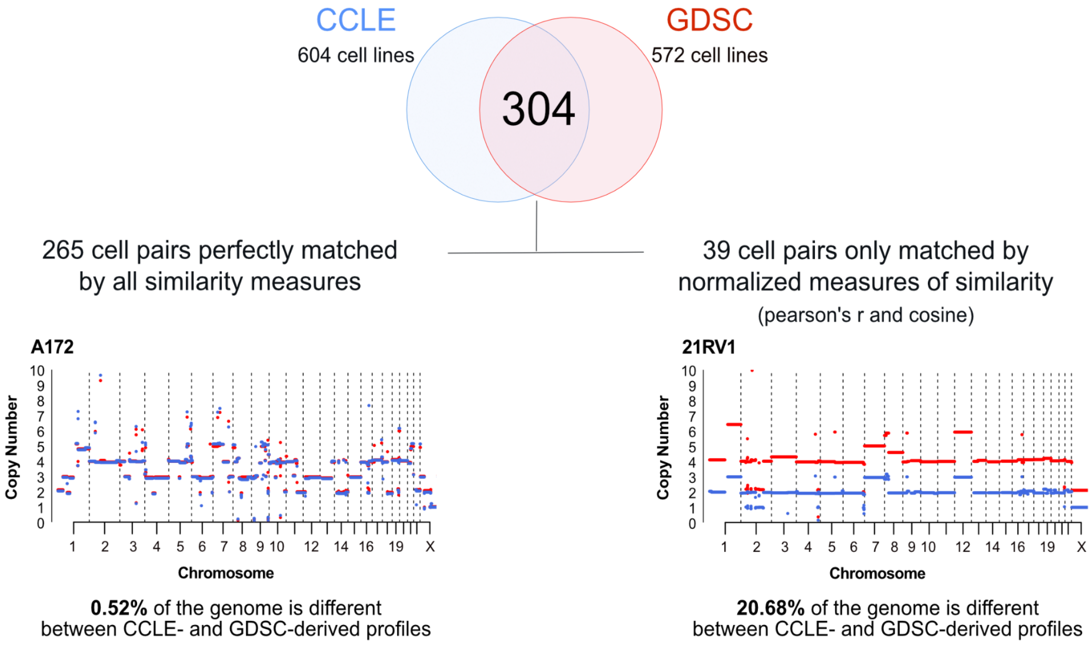

Supplementary Figure 1

Summary of CNpare performance when used to correctly match 304 GDSC cell lines within the CCLE database. In total, 265 of the 304 lines matched across all metrics. The copy number plot on the left shows an example of a GDSC culture cell line copy number profile (red) matched with its CCLE culture profile top hit (blue) across all similarity metrics. The copy number plot on the right is an example of one of the 39 cell line pairs which did not match for Manhattan and Euclidean distance (but did match for Pearson's r and Cosine similarity). In this case, these cell lines have different copy number ploidy fits but similar copy number changes. Under each plot, the normalized percentage of genome difference at copy number segment level between the cell pairs is indicated. The copy number profiles were adjusted for identifying ploidy differences for later estimating the percentage of difference by dividing the total length of the different segments by the whole genome size.

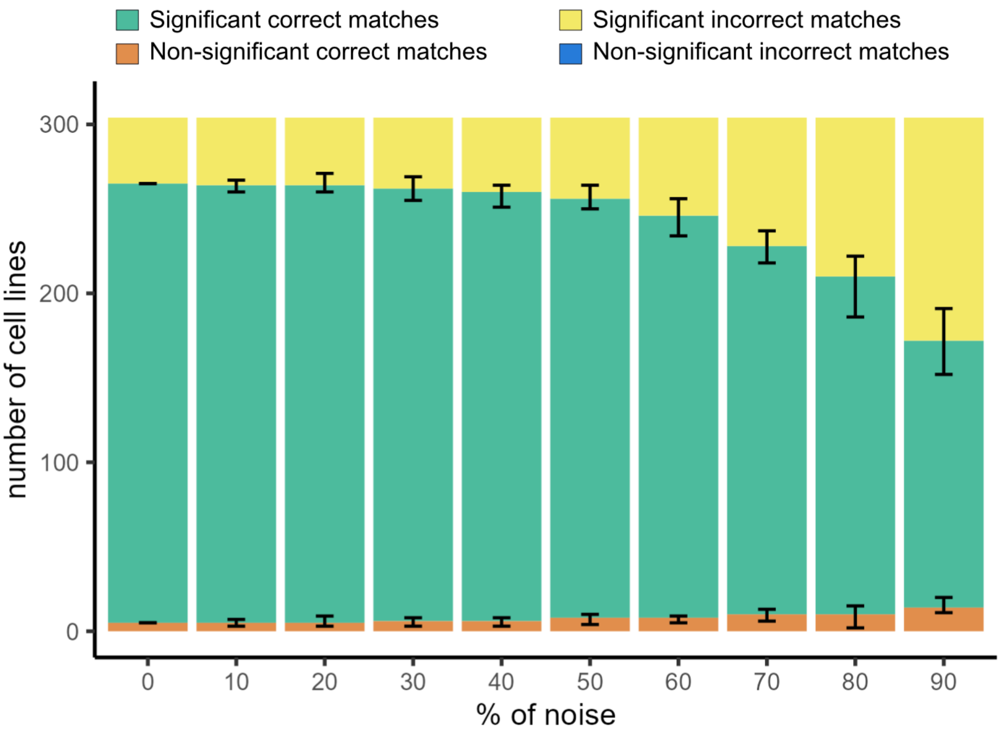

Supplementary Figure 2

Number of cell lines matched correctly and incorrectly after the introduction of noise in the copy number profiles from CCLE dataset. Noise was introduced by randomly perturbing different percentages of copy number segments. Bars represent the median from 10 simulations, with error bars showing the minimum and maximum. The Manhattan distance was used to identify, for each CCLE line perturbed, the matched cell line in the GDSC dataset. The empirical p-value was calculated to assess significance. Bars are coloured-coded to indicate statistical significance of the match.

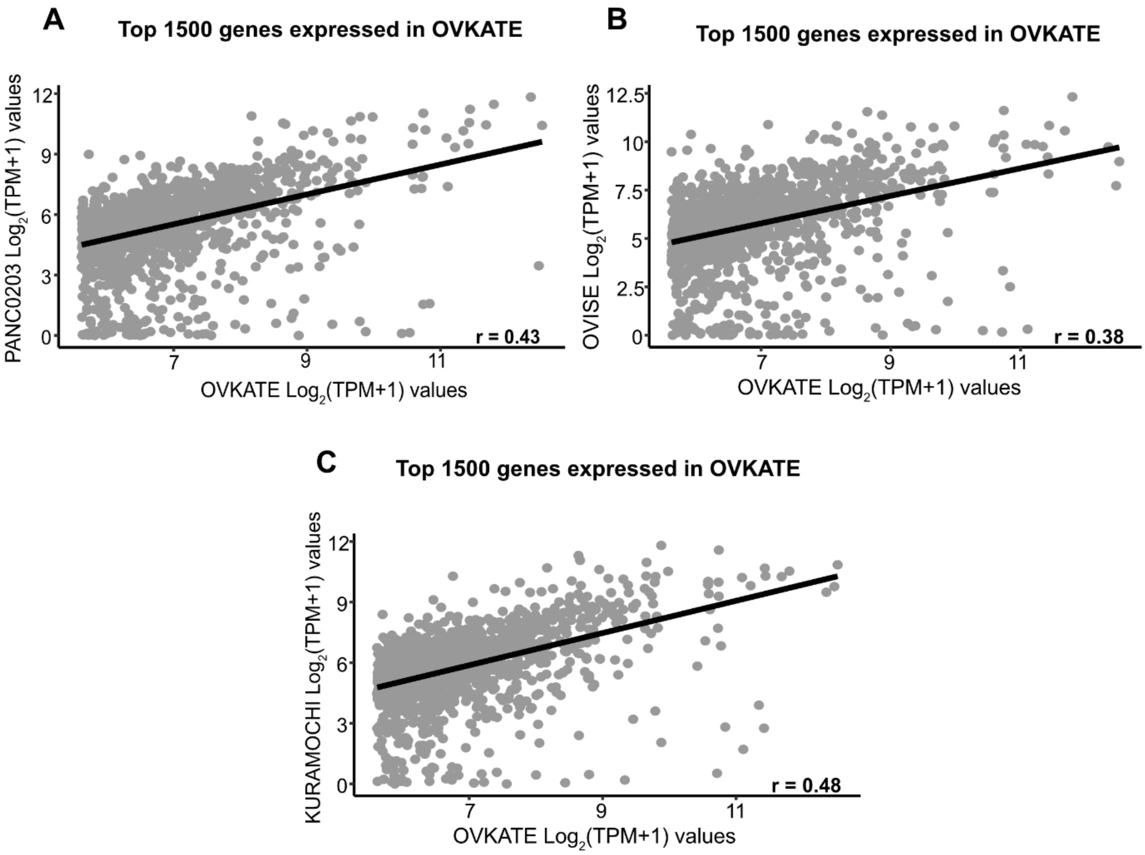

Supplementary Figure 3

Gene expression correlation between the OVKATE cell line and PANC0203 (A), OVISE (B) and KURAMOCHI (C). Only the 1,500 genes belonging to cancer-related pathways with the highest expression levels in OVKATE were analysed.

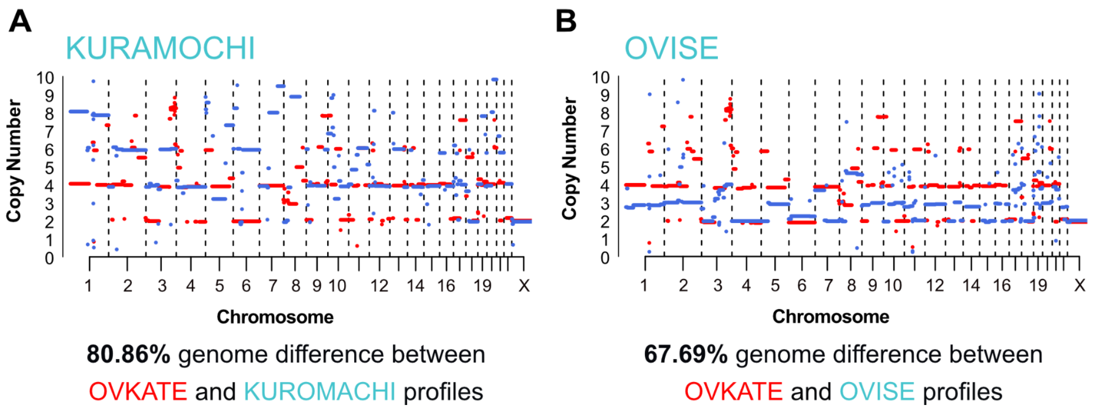

Supplementary Figure 4

Copy number plots showing the copy number profiles of OVKATE and the matching KURAMOCHI (**A**) and OVISE (**B**) cell lines based on copy number signature exposures clustering using cosine similarity

Supplementary Table 1

Similarity metrics	Properties	Recommendations
Pearson's r	Normalized-based metric<br>● Invariant to scaling, thus magnitude is ignored<br>● Invariant to location shifts of data values	Identification of similar profiles regardless of their ploidy status. Preferable to cosine similarity for the case of highly fragmented profiles
Cosine similarity	Normalized-based metric<br>● Invariant to scaling, thus magnitude is ignored<br>● Used for high-dimensional data	Identification of similar profiles regardless of their ploidy status
Manhattan distance	Magnitude-based metric<br>● Affected by the feature units<br>● Used for high-dimensional data	Identification of profiles with similar focal events and ploidy status. Preferable to euclidean distance for the case of highly fragmented profiles
Euclidean distance	Magnitude-based metric<br>● Affected by the feature units<br>● Used for low-dimensional data	Identification of profiles with similar focal events and ploidy status

Supplementary Table 2

**Bin resolution**	**Similarity metrics**			
	Pearson's r	Cosine similarity	Manhattan distance	Euclidean distance
100kb	304 (100%)	304 (100%)	265 (87.17%)	269 (88.49%)
500kb	304 (100%)	304 (100%)	265 (87.17%)	265 (87.17%)
1Mb	304 (100%)	304 (100%)	265 (87.17%)	265 (87.17%)

Supplementary methods

Data acquisition and curation

Precomputed absolute copy number profiles for cell lines appearing in the Cancer Cell Line Encyclopaedia (CCLE) (Ghandi *et al.*, 2019) project and the Genomics of Drug Sensitivity in cancer (GDSC) project (Yang *et al.*, 2013) were downloaded from https://github.com/VanLoo-lab/ASCAT.sc (Van Loo *et al.*, 2010). Only cell lines with sufficient evidence of chromosomal instability were included in the CNpare database. To compute this, we smoothed normal segments by collapsing and merging near diploid segments, and then removed samples with less than 20 non-diploid aberrations. This reduced the number of available cell line profiles to 1,171.

Data preprocessing

To enable comparison between two copy number profiles, segmented profiles were converted into evenly sized bins across the genome (default 500 kb). To adjust for noise at copy number boundaries, a window-based smoothing procedure was used to align boundaries across samples. CNpare also provides the option to match samples based on the exposure to different types of chromosomal instability. To do this, we computed signature activities for the 7 ovarian signatures from copy number profiles by applying the computational approach outlined in (Macintyre *et al.*, 2018). For running this approach, copy number data was initially formatted into a list of segment tables (one element of the list per sample). Then, values of six different features from each copy number profile were computed, and then the linear combination decomposition function from YAPSA(Schlesner, 2017) was used to compute the signature activities in each sample.

Comparison of copy number profiles

Using genome-wide copy number

Similarity between bin-level copy number profiles was quantified using four different metrics: Pearson correlation coefficient, Manhattan distance, Euclidean distance and Cosine Similarity. The Pearson correlation test was computed using the *cor()* function from the *stats* R package (version 3.6.2), while the other metrics were directly calculated. Although these four metrics report the level of similarity or difference, all of them do not necessarily report a similar hit and the most informative depends on the case in question. Distance-based metrics may be used for measuring similarity when the ploidy magnitude of the profiles matters. However, since Pearson correlation coefficient and Cosine Similarity are normalized measures, they can be used to identify similar profiles regardless of their ploidy magnitude (see Supplementary Table 1). Similarity values of all possible pairwise comparison tests on the CNpare database were used to calculate the empirical p-value. Empirical p-values were calculated by dividing the number of pairwise comparisons with a better similarity value than the one obtained, by the total number of comparisons made.

Using copy number signatures

Comparison based on copy number signatures was performed via clustering. In this case, CNpare reports similar cell lines based on shared cluster membership. k-means clustering was performed using the *akmeans()* function from the *akmeans* R package (version 1.1). This method automatically identifies the optimal number of clusters. Cosine similarity between the 7 copy number signatures was used as a distance metric. *plotClusters()* function was used for plotting clusters, coloring the cluster where samples obtained using *getClusterSamples()*.

Calculating percentage genome difference

Percentage genome difference was calculated at a copy number segment level and represents the fraction of the total human genome length where the segment value between the input profile and the matched profile is different. To compute this, copy number values of the input profile were recorded for each segment present in the matched cell line copy number profile. If more than one segment of the input profile fell within a reference segment, these segments were merged by computing the median. Copy numbers were rounded to the nearest integer for computing differences. The total length of different segments was divided

by the whole genome size, and the percentage reported. For normalised measures, the two copy number profiles were adjusted to account for ploidy differences before percentage genome difference was computed.

Robustness analysis

Assessing performance across different bin sizes

We assessed the performance of CNpare using three different bin sizes (100kb, 500kb and 1Mb). For the 304 cell lines profiled in the CCLE and GDSC projects, *segment tables* were converted to *bin tables* according to each bin size. Then, we compared the ability of CNpare to match cell line pairs across the different bin sizes by computing four similarity metrics: Pearson's r, Cosine Similarity, Manhattan distance and Euclidean distance. No significant differences in performance were observed across bin resolutions (Supplementary Table 3).

Assessing performance with noise via simulations

We tested the impact of noise on CNpare's ability to identify cell line pairs. Copy number noise typically manifests in two ways: 1) incorrect segmentation resulting in a shift in the copy number of a segment to a neighboring copy number state; 2) incorrect ploidy estimation. As our method overcomes noise in the ploidy estimation, we focused on noise in the segmentation. To include segmentation noise in the copy number profiles of CCLE cell lines we randomly shifted the copy number of segments to the neighboring copy number state by adding or subtracting 1 to the copy number value. We tested different perturbation levels (from 10% to 90% of the segments in a profile), and ran the simulation 10 times.

Then, we evaluated the ability of CNpare to identify, for each artificially perturbed CCLE line, the same cell line profiled in the GDSC project. To perform a ploidy aware comparison, we used the Manhattan distance for matching cell line pairs. Empirical p-values were calculated to assess deterioration of matches. We plotted the median of correct and incorrect matches coloured by significance, with the minimum and maximum taken from the 10 simulations (Supplementary Figure 2).

Comparing performance to other approaches

Gene-level copy number

Previous approaches that compared gene based copy number use the relative copy number of genes above or below a certain threshold (Barretina *et al.*, 2012; Ben-David *et al.*, 2018). To replicate this approach, gene positions were downloaded from UCSC Table Browser (Karolchik *et al.*, 2004) and copy number for each transcription start of 18,944 protein-coding genes was determined by transforming the absolute to relative copy number (log2 of the ratio of gene absolute copy number over the genome-wide median absolute copy number). Genes with a relative copy number greater than 0.3 or less than -0.3 were used for comparison (default threshold used by previous approaches). These gene-level copy numbers were compared for each cell line culture pair using Pearson correlation. The proportion of cell pairs correctly matched was 63% (190 cells). However, if the absolute copy number of each gene was used rather than relative with a threshold, then this proportion increased to 100%, mimicking the results obtained at genome-based level. This suggests, with sufficient number of bins sampled across the genome, performance can be achieved equivalent to whole-genome based comparisons.

Chromosome arm copy number

Similar to gene based comparisons, previous approaches for comparing chromosome arm-based copy number use relative copy number. We therefore computed the whole arm chromosome copy number by averaging the relative copy number values of all segments aligned to each chromosome arm(Ben-David *et al.*, 2018). We performed a Pearson correlation to compare culture pairs from the CCLE and GDSC databases. 91% (278 cells) of the cell-line pairs matched.

Ploidy status

For each cell line, we computed the weighted mean absolute copy number values of all segments to infer the overall ploidy. The relative weight of each segment depends on their length. Mean values were then rounded to one decimal point and the proportion of cell pairs matched by the ploidy status was then computed (43%, 131 cells).

Gene-expression profiles

RNAseq FPKM gene expression data from 73 cell lines included in both CCLE and GDSC were downloaded from Cell Model Passports (https://cellmodelpassports.sanger.ac.uk/downloads). Gene expression levels were compared across cell lines using Pearson correlation.

All the results of these comparisons are available at Supplementary Data 1.

Real world performance assessment

Next best matches for each cell line in database

We applied CNpare to identify the next best match for each of the 604 CCLE cell lines and quantified the frequency of significant matches (empirical p-value < 0.05). We performed this analysis using both a non-normalized (Manhattan distance) and a normalized (Pearson's r) similarity measure.

Testing suitability of OVKATE cell line matches

To validate the suitability of the cell lines matched with OVKATE, we performed a correlation analysis between their gene expression profiles. Log_2 transformed TPM gene expression data using a pseudocount of 1 (log_2(TPM+1)) for the protein coding genes were downloaded from DepMap Public 21Q3 (https://depmap.org/portal/download/). We only used genes appearing in the c6 dataset at msigDB. For performing the correlations, we further filtered this genelist to contain only the 1500 genes with the highest expression levels in the OVKATE cell line. Correlation between expression levels of OVKATE and the matched cell line was performed using Pearson correlation test. Empirical p-values were calculated by dividing the number of pairwise comparisons with a better similarity value than the one obtained, by the total number of comparisons made.

Software versions

The following packages were used under R version 4.0.2 (2020-06-22).

**Package's name**	**Version**	**Reference**
base	4.0.2	(R Core Team, 2020)
Biobase	2.50.0	(Huber *et al.*, 2015)
BiocGenerics	0.36.1	(Huber *et al.*, 2015)
cluster	2.1.0	(Maechler *et al.*, 2019)
data.table	1.14.0	(Dowle and Srinivasan, 2021)
datasets	4.0.2	(R Core Team, 2020)
doParallel	1.10.16	(Corporation and Weston, 2020)
dplyr	1.0.7	(Wickham *et al.*, 2021)
factoextra	1.0.7	(Kassambara and Mundt, 2020)
flexmix	2.3-17	(Grün and Leisch, 2008)
foreach	1.5.1	(Wallig and Weston, 2020)
ggplot2	3.3.5	(Wickham, 2016)
grDevices	4.0.2	(R Core Team, 2020)
graphics	4.0.2	(R Core Team, 2020)
iterators	1.0.13	(Analytics and Weston, 2020)
kableExtra	1.3.4	(Zhu, 2021)
lattice	0.20-41	(Sarkar, 2008)
magrittr	2.0.1	(Bache and Wickham, 2020)
methods	4.0.2	(R Core Team, 2020)
NMF	0.23.0	(Gaujoux and Seoighe, 2010)
parallel	4.0.2	(R Core Team, 2020)
pkgmaker	0.32.2	(Gaujoux, 2020)
QDNAseq	1.26.0	(Scheinin *et al.*, 2014)
qusage	2.24.0	(Meng *et al.*, 2019)
RColorBrewer	1.1-2	(Neuwirth, 2014)
registry	0.5-1	(Meyer, 2019)
reshape2	1.4.4	(Wickham and Others, 2007)

rngtools	1.5	(Gaujoux)
splitstackshape	1.4.8	(Mahto, 2019)
stringr	1.4.0	(Wickham, 2019)
stats	4.0.2	(R Core Team, 2020)
utils	4.0.2	(R Core Team, 2020)
YAPSA	1.16.0	(Schlesner, 2017)

References

Analytics,R. and Weston,S. (2020) iterators: Provides Iterator Construct.

Bache,S.M. and Wickham,H. (2020) magrittr: A Forward-Pipe Operator for R.

Barretina,J. *et al.* (2012) The Cancer Cell Line Encyclopedia enables predictive modelling of anticancer drug sensitivity. *Nature*, **483**, 603–607.

Ben-David,U. *et al.* (2018) Genetic and transcriptional evolution alters cancer cell line drug response. *Nature*, **560**, 325–330.

Corporation,M. and Weston,S. (2020) doParallel: Foreach Parallel Adaptor for the 'parallel' Package.

Dowle,M. and Srinivasan,A. (2021) data.table: Extension of `data.frame`.

Gaujoux,R. (2020) pkgmaker: Development Utilities for R Packages.

Gaujoux,R. Rngtools: Utility Functions for Working with Random Number Generators; R Package Version 1.5. 2020.

Gaujoux,R. and Seoighe,C. (2010) A flexible R package for nonnegative matrix factorization. *BMC Bioinformatics*, **11**, 367.

Ghandi,M. *et al.* (2019) Next-generation characterization of the Cancer Cell Line Encyclopedia. *Nature*, **569**, 503–508.

Grün,B. and Leisch,F. (2008) FlexMix Version 2: Finite Mixtures with Concomitant Variables and Varying and Constant Parameters. *Journal of Statistical Software*, **28**.

Huber,W. *et al.* (2015) Orchestrating high-throughput genomic analysis with Bioconductor. *Nat. Methods*, **12**, 115–121.

Karolchik,D. *et al.* (2004) The UCSC Table Browser data retrieval tool. *Nucleic Acids Res.*, **32**, D493–6.

Kassambara,A. and Mundt,F. (2020) factoextra: Extract and Visualize the Results of Multivariate Data Analyses.

Macintyre,G. *et al.* (2018) Copy number signatures and mutational processes in ovarian carcinoma. *Nat. Genet.*, **50**, 1262–1270.

Maechler,M. *et al.* (2019) cluster: Cluster Analysis Basics and Extensions.

Mahto,A. (2019) splitstackshape: Stack and reshape datasets after splitting concatenated values. *R package version*, **1**.

Meng,H. *et al.* (2019) Gene set meta-analysis with Quantitative Set Analysis for Gene Expression (QuSAGE). *PLoS Comput. Biol.*, **15**, e1006899.

Meyer,D. (2019) registry: Infrastructure for R Package Registries.

Neuwirth,E. (2014) RColorBrewer: ColorBrewer palettes. R package version 1.1-2.

R Core Team (2020) R: A Language and Environment for Statistical Computing.

Sarkar,D. (2008) Lattice: Multivariate Data Visualization with R Springer New York.

Scheinin,I. *et al.* (2014) DNA copy number analysis of fresh and formalin-fixed specimens by shallow whole-genome sequencing with identification and exclusion of problematic regions in the …. *Genome*.

Schlesner,D.H.Z.G. (2017) YAPSA Bioconductor.

Van Loo,P. *et al.* (2010) Allele-specific copy number analysis of tumors. *Proc. Natl. Acad.*

Sci. U. S. A., **107**, 16910–16915.
Wallig,M. and Weston,S. (2020) foreach: Provides Foreach Looping Construct.
Wickham,H. *et al.* (2021) dplyr: A Grammar of Data Manipulation.
Wickham,H. (2016) ggplot2: Elegant Graphics for Data Analysis.
Wickham,H. (2019) stringr: Simple, Consistent Wrappers for Common String Operations.
Wickham,H. and Others (2007) Reshaping data with the reshape package. *J. Stat. Softw.*, **21**, 1–20.
Yang,W. *et al.* (2013) Genomics of Drug Sensitivity in Cancer (GDSC): a resource for therapeutic biomarker discovery in cancer cells. *Nucleic Acids Res.*, **41**, D955–61.
Zhu,H. (2021) kableExtra: Construct Complex Table with ‘kable’ and Pipe Syntax.
